# Aberrant Splicing Events Associated to *CDH23* Noncanonical Splice Site Mutations in a Proband with Atypical Usher Syndrome 1

**DOI:** 10.3390/genes10100732

**Published:** 2019-09-21

**Authors:** Rebeca Valero, Marta de Castro-Miró, Sofía Jiménez-Ochoa, Juan José Rodríguez-Ezcurra, Gemma Marfany, Roser Gonzàlez-Duarte

**Affiliations:** 1 DBGen Ocular Genomics, 08028 Barcelona, Spain; rvalero@ub.edu (R.V.); martadecastromiro@ub.edu (M.d.C.-M.); sofiacorella96@gmail.com (S.J.-O.); gmarfany@ub.edu (G.M.); 2 Departament de Genètica, Microbiologia i Estadística, Facultat de Biologia, Universitat de Barcelona, Avda.Diagonal 643, 08028 Barcelona, Spain; 3 Barraquer—Centro de Oftalmología Barcelona, 08021 Barcelona, Spain; jrezcurra@barraquer.com; 4 Centro de Investigación Biomédica en Red en Enfermedades Raras (CIBERER), 08025 Barcelona, Spain; 5 Institut de Biomedicina de la Universitat de Barcelona-Institut de Recerca Sant Joan de Deu (IBUB-IRSJD), 08028 Barcelona, Spain

**Keywords:** Usher syndrome, *CDH23*, splice site variants, functional analysis, phenotypic effects

## Abstract

Aims: The aim of this study was the genetic diagnosis by next generation sequencing (NGS) of a patient diagnosed with Usher syndrome type 2 and the functional evaluation of the identified genetic variants to establish a phenotype–genotype correlation. Methods: Whole exome sequencing (WES) analysis identified two heterozygous intronic variants in *CDH23*, a gene responsible of Usher syndrome type 1. Evaluation of the putative splicing effects was performed in vivo, in whole blood samples, and in vitro, by transfection of midigene constructs in HEK293T cells. Results: Two intronic variants were identified in intron 45 of *CDH23*—one novel, c.6050-15G>A, and the other, c.6050-9G>A, already reported as a noncanonical splice site (NCSS) mutation—with partial functional characterization. In vivo and in vitro analyses showed aberrant transcripts by the addition of 13 and 7 nucleotides to exon 46, respectively. Transcript degradation by nonsense mediated decay (NMD) in blood cells could only be prevented by cycloheximide treatment. Midigene constructs showed that the two variants contributed to exon skipping and generated aberrantly spliced transcripts. Conclusions: A combination of in vivo and in vitro assays provided a comprehensive view of the physiological effects of NCSS variants, which in this case led to a clinical reassignment of the proband as affected with atypical USH1 syndrome.

## 1. Introduction

Usher syndrome (USH) is a group of inherited autosomal recessive disorders characterized by partial or total progressive hearing and vision loss. All Usher disease forms are clinically and genetically heterogeneous and constitute the most common cause of combined deafness and blindness [1]. Sensorineural hearing impairment is caused by abnormalities of the inner ear, and vision loss occurs as photoreceptor cells in the retina deteriorate progressively, leading to retinitis pigmentosa (RP) [2].

Three major clinical USH subtypes are distinguished: Type I (USH1), Type II (USH2), and Type III (USH3), on the basis of the severity of the clinical traits [1,3]. USH1 represents the most severe form, showing extensive genetic and allelic heterogeneity, and is characterized by severe to profound congenital deafness, variable vestibular areflexia, and adolescent onset retinitis pigmentosa [4]. To date, six genes have been identified as causative of USH1: *MYO7A, USH1C, CDH23, PCDH15, USH1G*, and *CIB2*. Of those, the major gene associated with USH1 is *MYO7A*, which accounts for up to 70% of cases [5,6,7]. Usher Type II (USH2) is associated with moderate to severe hearing loss, absence of vestibular dysfunction, and later onset retinal degeneration. Three USH2 causative genes have been identified. Of these, the USH2A gene explains the majority of cases (85–86%) whereas *GPR98* (USH2C) and *DFNB31* (USH2D) account for the remainder (15–14%) [7,8]. Usher Type III, mainly caused by mutations in *CLRN1* (USH3A), shows progressive hearing loss, variable onset of RP, and vestibular response. USH3 is rare, except in the Finnish population and among Ashkenazi Jews [9].

*CDH23*, a member of the cadherin superfamily, maps to chromosome 10, encompasses more than 290 kb and 69 exons, and encodes a 3.354 amino acid protein. Cadherin family genes encode integral membrane proteins that mediate calcium-dependent cell–cell adhesion. *CDH23* plays an essential role in maintaining normal retinal and cochlear function. A total of 360 different *CDH23* mutations have been described to date, revealing an interesting genotype–phenotype correlation [10,11,12]. Protein truncating mutations in *CDH23* due to nonsense, frameshift, or splice site pathogenic variants cause USH1, whereas missense mutations in the same gene usually are causative of non-syndromic deafness [12]. In addition, a missense mutation c.4136G > T has been associated to familial and sporadic pituitary adenomas [13]. Notably, more than 40 (>10%) of the reported pathogenic mutations in *CDH23* alter the splicing pattern of the gene (an updated list of *CDH23* splicing mutations is shown in Table 1).

In rare diseases, most pathogenic variants that disrupt splicing alter canonical donor or acceptor consensus splice sites (DS and AS, respectively), but other variants located at the flanking regions, in the so-called noncanonical splice sites (NCSS), are also emerging as causative of aberrant splicing events. NCSS variants, whether pathogenic or not, are usually detected either by direct sequencing of gene exons or after target gene and next-generation sequencing. Most intronic NCSS variants are located either at positions +3 to +6 downstream DSs, or −14 to −3 upstream ADs, including the polypyrimidine tract) [15], and are often classified as either variants of unknown significance (VUS) or non-disease-causing variants [16]. Although *in silico* algorithms can be used to assess the possible effect of the identified changes in splicing, the results of such tests are only predictive, and the precise effect of the specific mutation should be verified in functional studies. Among the potential molecular consequences of the NCSS variants, the production of aberrant transcripts due to exon skipping and/or frameshift would result in nonsense-mediated decay of transcripts bearing a premature stop codon. In fact, it has been estimated that about one third of disease-associated mutations alter pre-mRNA splicing [17], thus underscoring the importance of in vivo/in vitro splice assays to test and verify the pathogenicity of these types of nucleotide variants.

Here, we report the identification and in vivo characterization of a novel NCSS mutation in *CDH23* (c.6050-15G>A) in double heterozygous combination with a previously reported NCSS in the same intron c.6050-9G>A mutation [18] by direct RT-PCR from fresh whole blood samples. This useful method allows the rapid and direct evaluation of the effect of VUS variants in the splicing of genes that are expressed in this accessible tissue. In addition, the effects on splicing of each allele have been individually assessed and further confirmed by in vitro splicing assays.

## 2. Materials and Methods 

### 2.1. Clinical Diagnosis 

An adult female patient was clinically diagnosed with Usher syndrome on the basis of ophthalmic studies that included visual acuity, visual field, fundus ophthalmoscopy, electroretinography, pure-tone audiometry, and vestibular evaluation. Because of the absence of vestibular dysfunction, the patient was preliminarily classified as USH2 type, pending confirmation by genetic diagnosis. Consent for genetic testing was obtained from the patient and family.

### 2.2. Samples

Peripheral blood DNA from patient and available relatives (parents and unaffected brother) were obtained using the QIAamp DNA Blood Maxi Kit (Qiagen, Hilden, Germany). After approval from the Bioethics Committee of the Universitat de Barcelona (Institutional Review Board IRB_00003099, 2016), written informed consents from all the individuals were obtained following the tenets of the Declaration of Helsinki prior to donation of blood samples. The DNA from the patient was analyzed by whole exome sequencing (WES). Variants identified in any gene associated with USH were carefully prioritized by allele frequency in gnomAD and our cohort of controls, as well as the predicted molecular phenotypic effect. Only *CDH23* showed two variants located in either exonic sequences or adjacent to the splicing sites, one of them being a reported causative mutation. The two *CDH23* variants were confirmed by Sanger sequencing. Cosegregation analysis in the family was performed by specific PCR followed by Sanger sequencing.

### 2.3. In Silico Analysis of the Effect of Variants in Splicing 

The potential effect of the non-canonical splice variants c.6050-15G>A and c.6050-9G>A on the splicing of *CDH23* was assessed comparing the wild-type and mutated sequences using four different algorithms (SpliceSite Finder, MaxEntScan, NNSPLICE, and GeneSplicer) via Alamut Genova Software, http:///www.interactive-biosoftware).

### 2.4. In Vivo Splicing Analysis of CDH23 Transcripts in the Patient

After previous confirmation that *CDH23* was expressed in blood, samples from the patient and control were used to analyze the splicing pattern of exons 45-46-47 by RT-PCR. Cycloheximide was added to fresh blood samples to avoid nonsense-mediated decay of aberrant out-of-frame transcripts. Duplicate samples were obtained to compare untreated versus treated samples (cycloheximide at 100 µg/ml, incubation for 4 h at RT). After 4 h, RNAlater solution (ThermoFisher Scientific, Waltham, MA, USA) was added to both treated and untreated duplicates, following the manufacturer’s instructions. Total RNA was extracted using RiboPure-Blood Kit (Life Technologies, now ThermoFisher Scientific, Waltham, MA, USA) according to the manufacturer’s instructions. First, cDNA strand was synthesized using the qScript cDNA Synthesis Kit (QIAGEN, Hilden, Germany). For the analysis of splicing events, first-strand cDNA templates were used to amplify the region of interest (exons 45-46-47 of *CDH23*) with specific primers (Exon45F: 5′-CCTCTCACGGTGCTCAATGG-3′, Exon47R: 5′-CAAAGGCGTCCTCCTGGTTG-3′). The amplification reaction consisted of 40 cycles (94 °C for 30 s, 62 °C for 30 s, and 72 °C for 30 s). PCR products were visualized on 1.5% agarose gels stained with SYBR Safe DNA Gel Stain (ThermoFisher Scientific, Waltham, MA, USA) and sequenced. RT-PCR products were also subcloned into pGEM-T-Easy vector (Promega, Madison, WI, USA) and transformed into DH5α cells. Positive clones were analyzed by Sanger sequencing.

### 2.5. In Vitro Splicing Assays in HEK293T Cells

For individual analysis of each of the *CDH23* variants, (c.6050-15G>A and c.6050-9G>A), genomic fragments of 3751bp that spanned exons 45, 46, and 47 were subcloned into the HIV-tat intron of the pSPL3 expression vector (Addgene, Watertown, MA, USA). HEK293T cells were seeded on 24-well plates (50,000 cells/well) and grown in DMEM (Thermofisher Scientific, Waltham, MA, USA) supplemented with 10% of FBS (fetal bovine serum). After 24 h, the cells were lipofected with constructs bearing either the pSPL3-wtCDH23 midigene (MGC1-WT), pSPL3-mutCDH23 midigene (containing the c.6050-9G>A or c.6050-15G>A variants (MGC1-9A and MGC1-15A, respectively)), or the empty pSPL3 vector. Transfections were performed according to the manufacturer’s instructions (Lipotransfectin, Guillena, Spain). Thirty hours after transfection, cells were treated with cycloheximide (100 ug/ml) for 4 h. After 4 h treatment, untreated and treated cells were collected and lysed and total mRNA was obtained using a High Pure RNA Isolation Kit (Roche Life Sciences, Indianapolis, IN, USA). First, cDNA strand and RT-PCR of the *CDH23* region of interest were performed, as detailed above, for the in vivo analysis of transcripts. Amplified bands were directly excised from the gel, purified, and analyzed by Sanger sequencing.

## 3. Results

**Figure 1:**
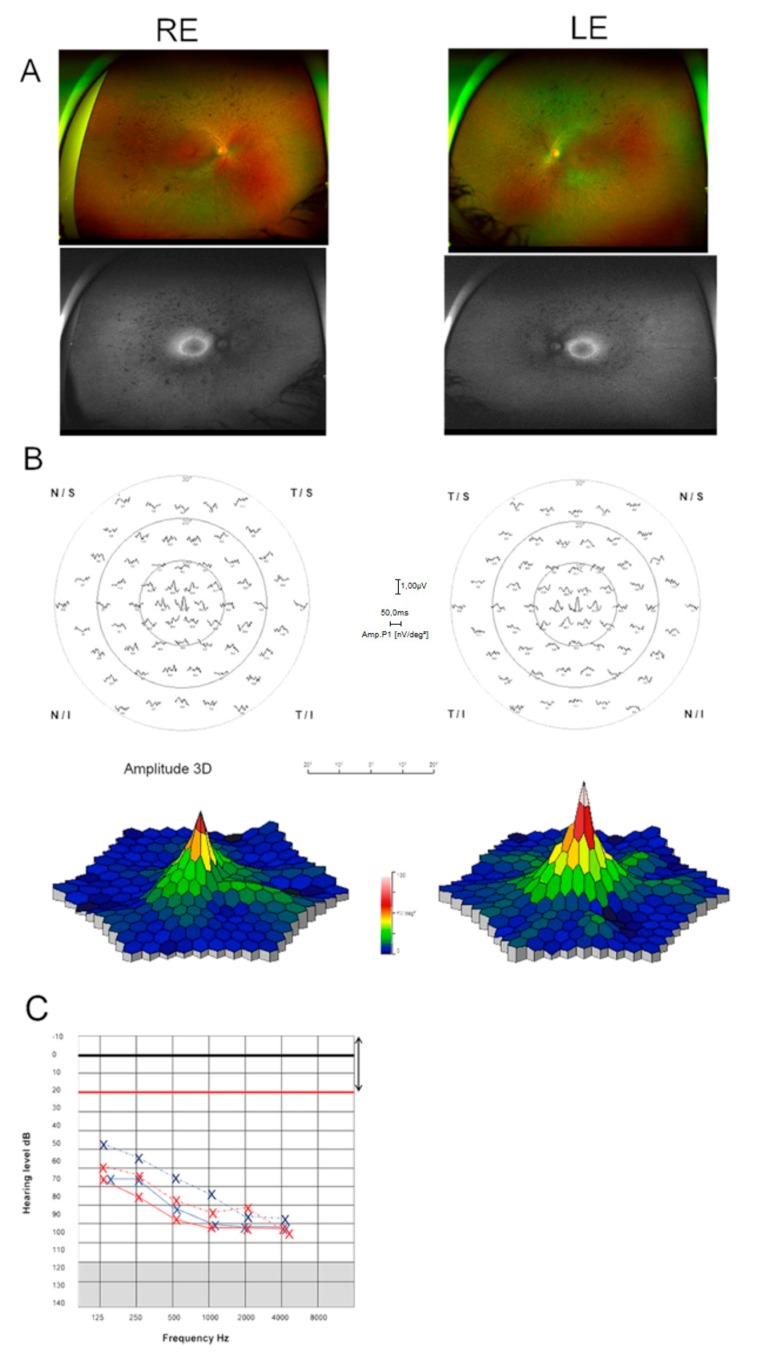
Clinical findings of patient 96DBG1 diagnosed with Usher syndrome. (**A**) Eye fundus of the right eye (RE) and left eye (LE) at 29 years of age, showing pigment peripheral deposits, vascular attenuation, and pallor of the optic nerve. Autofluorescent images show a hyperfluorescent ring in the perimacular region. (**B**) Multifocal electroretinogram (ERG) plots of the right (RE) and left (LE) eyes with the three-dimensional (3D) representation showing concentric visual loss with preserved vison in the central 10 degrees area. (**C**) Audiometry scores of tests performed at 5 years old (dotted lines) and at 29 years old (straight lines), right ear—red, and left ear—blue.

The patient, an adult woman, reported congenital deafness without vestibular dysfunction. She was using hearing aids that allowed limited verbal communication. She reported night blindness since adolescence. Eye fundus examination showed pigment deposits in the medial peripheral area, vascular attenuation, and pallor of the optic nerve (Figure 1A, top panels). Tomographic and autofluorescence images revealed normal macular and foveal thickness and a discrete hyperfluorescent ring in the perimacular region (Figure 1A, bottom panels). Color vision was normal with altered contrast adaptation. Visual field tests and three dimensional-electroretinogram (3D-ERGs) recordings showed loss of peripheral concentric vision preserving the central 10 degrees (Figure 1B). All these traits are associated with retinitis pigmentosa. The ophthalmological examination showed that the clinical phenotype of the patient has remained stable for the last three years. The comparison of the audiometry tests recorded at 5 years old (1995) and 2019 showed stable congenital severe hearing loss (Figure 1C). The initial clinical diagnosis was Usher syndrome type II, and genetic diagnosis of the patient and family was required for confirmation.

**Figure 2:**
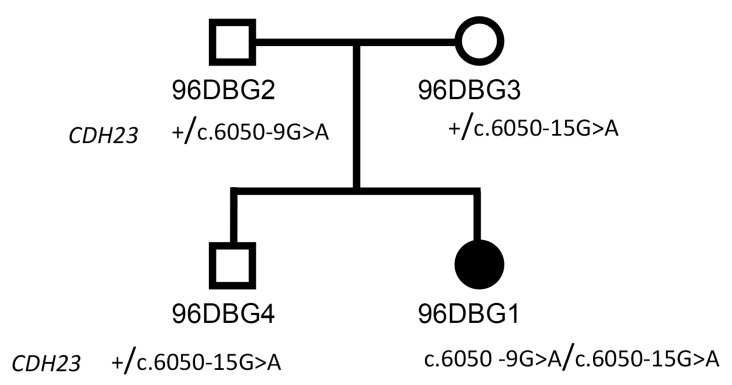
Pedigree of family 96DBG with the segregation analysis of *CDH23* variants.

After WES analysis and prioritization of genetic variants, two nucleotide variants were identified in the *CDH23* gene: c.6050-9G>A (already reported as pathogenic in an Usher syndrome patient [18]) and c.6050-15G>A (novel). The two identified genetic variants were validated by Sanger sequencing and confirmed by segregation analysis in the family (Figure 2). Mutations in *CDH23* have already been reported as causative of both typical and atypical Usher type I. The typical form is characterized by severe hearing loss, vestibular dysfunction, and retinitis pigmentosa; when one of these traits is not present, the disorder is clinically classified as atypical USH1 [4].

The variant c.6050-9G>A had been previously reported to alter the splicing of exon 46, therefore, we surmised that the new variant was also pathogenic and causative of aberrant splicing. To validate this hypothesis, several prediction algorithms (SSF, MaxEnt, NNSPLICE, GeneSplicer) were used to assess the potential splicing effects of the identified variants (c.6050-15G>A and c.6050-9G>A) compared to the wild-type *CDH23* sequence. Notably, only one of these four algorithms (MaxEnt) predicted the position of the wild-type acceptor site (AS), indicating that this sequence is not a consensus AS motif. Furthermore, the two identified genetic variants introduced putative novel acceptor sites, which were predicted even with higher score values than the wild-type sequence (Table 2). These in silico results prompted us to check whether the *CDH23* two alleles were indeed pathogenic and altered the splicing pattern.

Considering that most pathogenic variants affecting the noncanonical AS sequence caused either exon skipping or the inclusion of additional nucleotides, the potential effects of these identified intron 45 variants might cause an in-frame skipping of exon 46, or the addition of 7 and 13 nucleotides in exon 46. Both additions would result in a frameshift, leading to premature protein truncation and subsequent transcript degradation by nonsense-mediated decay (NMD). Therefore, total whole blood samples from the proband and control were analyzed after treatment with cycloheximide (CHX) to inhibit NMD. 

The variant c.6050-9G >A had been previously reported to alter the splicing of exon 46, therefore, we surmised that the new variant was also pathogenic and causative of aberrant splicing. To validate this hypothesis, several prediction algorithms (SSF, MaxEnt, NNSPLICE, GeneSplicer) were used to assess the potential splicing effects of the identified variants (c.6050-15G>A and c.6050-9G>A) compared to the wild-type *CDH23* sequence. Notably, only one of these four algorithms (MaxEnt) predicted the position of the wild-type acceptor site (AS), indicating that this sequence is not a consensus AS motif. Furthermore, the two identified genetic variants introduced putative novel acceptor sites, which were predicted even with higher score values than the wild-type sequence (Table 2). These in silico results prompted us to check whether the *CDH23* two alleles were indeed pathogenic and altered the splicing pattern.

Considering that most pathogenic variants affecting the noncanonical AS sequence caused either exon skipping or the inclusion of additional nucleotides, the potential effects of these identified intron 45 variants might cause an in-frame skipping of exon 46, or the addition of 7 and 13 nucleotides in exon 46. Both additions would result in a frameshift, leading to premature protein truncation and subsequent transcript degradation by nonsense-mediated decay (NMD). Therefore, total whole blood samples from the proband and control were analyzed after treatment with cycloheximide (CHX) to inhibit NMD. 

**Figure 3:**
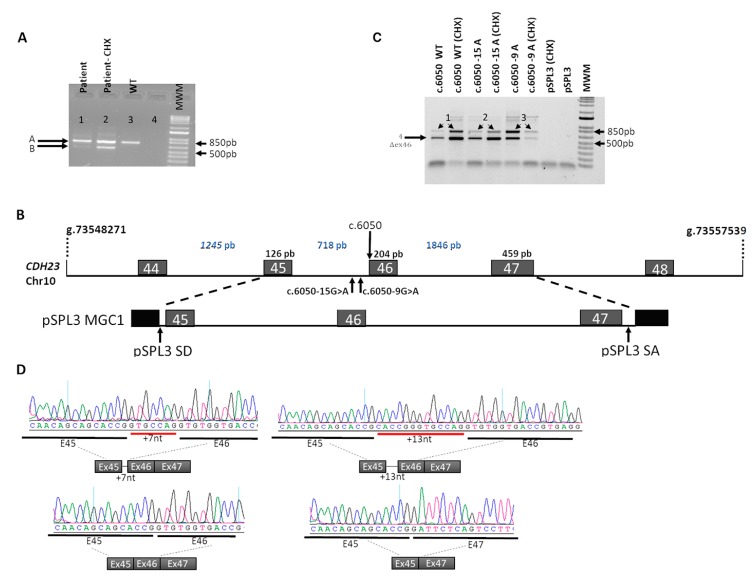
In vivo and in vitro splicing analysis of the identified *CDH23* alleles. (**A**) Analysis of the *CDH23* mRNAs of whole blood samples (treated and untreated with cycloheximide, CHX) from patient and control by RT-PCR, showing the presence of aberrantly spliced *CDH23* transcripts in addition to the wild-type transcript. Lane 1—patient sample (−CHX), Lane 2—patient sample (+CHX), Lane 3—control sample (−CHX), Lane 4—negative control. The band of 563 bp corresponded to the transcript with an in-frame skipping of exon 46. The band around 760–780 bp corresponded to the wild-type as well as to the aberrant transcripts with additional +7 and +13 nucleotides. Subsequent Sanger sequencing of cloned individual bands confirmed the insertion of 7 bp and 13 bp (774 pb, 780 pb bands) corresponding to each mutant allele. Transcripts bearing premature stop codon due to frameshift were only amplified in cycloheximide-treated blood samples (+CHX). (**B**) Schematic representation of the studied genomic region of *CDH23* gene and the midigene construct for in vitro splicing assays (MGC1). The position of each mutation is indicated. The genomic region encompassing exons 45, 46, and 47 was cloned between the splice donor (SD) and acceptor (SA) sites within the pSPL3 vector. (**C**) In vitro splicing assays in HEK293T cells transfected with either the wildtype (WT) or mutant *CDH23* midigenes (MGC1-WT, MGC1-15A, and MGC1-9A, respectively) with or without cycloheximide treatment (+CHX). All constructs (MGC1-WT, MGC1-15A, and MGC1-9A) produced skipping of exon 46. CHX treatment in cells transfected with the mutant constructs increased the relative amplification of the aberrantly spliced transcripts. (**D**) Sanger sequence analysis of each transcript band confirmed the wild-type splicing event in cells transfected with MGC1-WT, in contrast to the addition of +13 and +7 nucleotides in exon 46 in cells transfected with MGC1-15A and MGC1-9A, respectively.

Whole blood RT-PCR amplification of *CDH23* 45 to 47 exons (Figure 3A) produced one clear band corresponding to the wild-type transcript in the control (Figure 3A, lane 3). In contrast, two different products were amplified in the proband (Figure 3A, lanes 1 and 2), which became more evident after cycloheximide treatment—a shortest band that showed the size expected for exon 46 skipping (Figure 3A, band B) and a slower migrating band whose size was similar to that of the control (Figure 3A, band A). Direct sequencing of the PCR bands produced overlapping sequences from the splicing junction (Figure S1). Therefore, the PCR bands from CHX-treated samples were separately cloned and sequenced, confirming that the two variants did indeed produce aberrant transcripts (Figure S1). The G>A transition variant (c.6050-15G>A) generated a novel 15 bp upstream AS motif (AGCA) in intron 45, resulting in an addition of 13 bp in exon 46. This frameshift mutation would generate a truncated protein of 2029 amino acids (p.Gly2017Alafs*16). Aside from this, the variant c.6050-9G>A also created a novel acceptor site of exon 46, resulting in the frameshift addition of 7 bp in exon 46, in accordance with previous data [18]. These results confirmed the presence of nonsense-mediated decay (NMD) of the two aberrant transcripts, as they could only be amplified in cycloheximide-treated samples (Figure 3B, lanes 1 and 2). Of note, we also obtained amplification of wild-type spliced isoform in both cycloheximide-treated and -untreated samples from the proband, indicating that, at least in blood, some *CDH23* transcripts included the correct splicing of exon 46, suggesting that some amount of wild-type protein could still be produced.

In order to assess the contribution of each allele to the splicing isoforms observed in the patient, we performed in vitro exon-trapping assays. Wild-type (WT) or mutant midigene *CDH23* constructs were generated (MGC1-WT, MGC1-15A, MGC1-9A) and tested in transiently transfected HEK293T cells (Figure 3B). Midigenes contain the genomic context of the genetic variant, and in this case genomic fragments spanning from exon 45 to exon 47 were amplified from the proband DNA and cloned in a suitable exon-trapping vector (pSPL3), widely used for these type of assays. Endogenous *CDH23* expression in HEK293T cells was previously ruled out by RT-PCR analysis using specific *CDH23* primers. To avoid nonsense-mediated mRNA decay, cells were incubated with cycloheximide (details in the Materials and Methods section) (Figure 3C). The empty pSPL3 vector was used as negative control.

Sanger sequence analysis of the RT-PCR bands derived from MGC1-WT, MGC1-15A, or MGC1-9A transfected cells revealed two bands of different sizes. The shortest band corresponded to the transcript with skipping of exon 46 (Figure 3C, Δex46 band). On the other hand, the largest band (Figure 3C, bands 2 and 3) from cells transfected with MGC1-15A and MGC1-9A produced, respectively, the expected 780 bp and 774 bp transcripts containing the 13 bp and 7 bp added to exon 46, in accordance to the in vivo results (sequences in Figure 3D). However, compared to the in vivo analysis, NMD of frameshift transcripts was not clearly detected in transfected cells (the same results in CHX-treated and untreated cells), neither were we able to detect the wild-type transcript encompassing exons 45-46-47 in cells transfected with MGC1-15A and MGC1-9A.

## 4. Discussion

In order to secure the clinical diagnosis and provide an accurate prognosis for people affected with Usher syndrome, it is essential to identify the causative gene and assess the functional consequences of the nucleotide variants, particularly intronic variants, which can modulate the phenotypic severity depending on the combination of alleles. The phenotypic impact of non-coding variants in some diseases has already been illustrated by mutations in *ABCA4,* the major gene of Stargardt disease, where the functional analysis of NCSS and deep intronic variants have been crucial for the molecular diagnosis of unsolved cases and drawing genotype–phenotype correlations [19,20,21].

In this study, we identified two *CDH23* noncanonical splice site (NCSS) variants in an Usher syndrome patient, one of them being novel (c.6050-15G>A). Many mutations altering the splicing of *CDH23*, both in canonical and noncanonical splice sites, were reported, associated with an ample range of phenotypic severity of USH1 (a complete list of intronic *CDH23* mutations can be found in Table 1). The pathogenicity of the two identified NCSS variants in our proband was confirmed after analysis of the *CDH23* splicing pattern in vivo and in vitro. Canonical splice site mutations usually resulted in exon skipping, while the associated molecular effects of NCSS variants depended on the sequence alteration, the genomic context, and tissue-specific splicing factors [15]. In this work, bioinformatic analysis of the two *CDH23* NCSS variants predicted a shift in the AS splice site, and suggested their potential pathogenic effect. It is remarkable that the two nucleotide variants identified were located in the same intronic sequence and shifted the AS position, suggesting that the intron 45 AS is not a strong splicing motif, in accordance with the fact that most prediction algorithms miss the canonical WT AS.

The in vivo functional analysis of both *CDH23* intron 45 variants (c.6050-9G>A and c.6050-15G>A) showed not only the production of aberrantly spliced products (thus confirming the pathogenicity of the NCSS variants), but also the presence of relatively low levels of WT transcript (exons 45-46-47). Variable proportions of mutant and normal transcripts have been reported to be tissue-dependent in atypical Usher syndrome type I patients [22], and thus the splicing of *CDH23* is likely to show different regulation in whole blood and cochlea or retina, consequently explaining the milder phenotype (non-progressive RP and no vestibular dysfunction) of the proband, altogether leading to the reassignation of the clinical entity from USH2 into atypical USH1. 

The in vivo functional assays in whole blood of the proband confirmed the NMD of aberrantly spliced products of c.6050-9G>A and c.6050-15G>A *CDH23* variants, as expected for frameshift-causing mutations, since they could only be detected after cycloheximide treatment. In addition to the aberrant inclusion of extra nucleotides, the functional in vitro assays with *CDH23* midigene constructs were used to confirm the contribution of each individual mutation to the skipping of exon 46. Our results in transfected cells also supported the weakness of the WT AS of exon 46, since the control WT construct mainly produced the exon 46-skipped form, and very low levels of the correctly spliced transcript could be detected. In fact, the transcript with exon 46 skipping was in frame and may be of physiological value. Of note, exon 46 skipping has also been reported in patients carrying a mutation in the NCSS of the DS of exon 45 [14]. We believe that the combination of in vivo and in vitro assays, as those performed in our work, gives a more complete view of the physiological consequences of NCSS variants. NMD-mediated degradation of aberrant transcripts has only been detected using in vivo assays, whereas the individual contribution of each allele to altered splicing events could be evaluated by single construct transfections in vitro. Indeed, in vivo transcriptional analysis is restricted to cases where the gene of interest is expressed in whole blood or amenable tissues. Nonetheless, it is also worth considering that the relative amounts of differently spliced forms might shift in specific organs/tissues, such as the retina or inner ear. In fact, a tissue-specific splicing effect in the inner ear vs retina of a NCSS *MYO7A* (another Usher type I causative gene) mutation (c.5742+5G>A) has been reported in a mouse animal model [23], overall supporting the complexity of the phenotypic spectrum of the NCSS mutations.

## Figures and Tables

**Table 1:** Splicing mutations described in the *CDH23* gene, with their nucleotide position and associated phenotype (from the Human Gene Mutation Database, updated with mutations in [14], highlighting the mutations in intron IVS45 and those identified in this work (in red)).

Mutation	GRCh37/hg19	Intron	Disease
c.145+6T>G	73206158	IVS-2	Usher syndrome 1
c.288+1G>C	73269982	IVS-3	Usher syndrome 1
c.336+1G>A	73270759	IVS-4	Usher syndrome 1d
c.429+4G>A	73270973	IVS-5	Non-syndromic autosomal recessive deafness
c.1134+1G>A	73377151	IVS-10	Usher syndrome 1
c.1135-1G>T	73403617	IVS-10	Hearing loss
c.1987-2A>C	73447402	IVS-17	Usher syndrome 1
c.2176+1G>C	73450342	IVS-19	Deafness, non-syndromic, autosomal recessive
c.2177-2A>G	73453902	IVS-19	Usher syndrome 1
c.2289+1G>A	73454017	IVS-20	Usher syndrome 1d
c.2289+6T>G	73454022	IVS-20	Hearing loss, non-syndromic
c.2398-1G>T	73461778	IVS-21	Retinal disease
c.2587+1G>T	73461969	IVS-22	Usher syndrome 1
c.3580-1G>T	73490225	IVS-29	Usher syndrome 1
c.4104+4A>T	73492136	IVS-31	Usher syndrome 1
c.4105-4_4105-2delGCAinsTCT	73493993	IVS-31	Usher syndrome
c.4489-2A>C	73500577	IVS-35	Usher syndrome
c.4846-3C>G	73537434	IVS-37	Hearing loss, autosomal recessive
c.5068-2A>T	73537944	IVS-38	Usher syndrome 1
c.5187+2T>C	73538067	IVS-39	Usher syndrome 1
c.5368+1G>A	73539205	IVS-40	Usher syndrome 1
c.5820+5G>A	73545500	IVS-43	Sector retinitis pigmentosa and hearing loss
c.5821-2A>G	73548695	IVS-43	Usher syndrome 1
c.5923+1G>A	73548800	IVS-44	Usher syndrome
c.5924-2A>C	73550043	IVS-44	Hearing loss
c.6049G>A	73550170	E-44 NCSS IVS-45	Usher syndrome 1
c.6049+1G>A	73550171	IVS-45	Usher syndrome 1
c.6050-1G>C	73550888	IVS-45	Usher syndrome 1
c.6050-9G>A	73550880	IVS-45	Usher syndrome 1
c.6050-15>A	73550874	IVS-45	Usher syndrome 1
c.6712+1G>A	73553398	IVS-47	Usher syndrome 1
c.6829+1G>A	73556978	IVS-48	Usher syndrome 1
c.6829+2T>C	73556979	IVS-48	Usher syndrome 1
c.6830-2_6830delAGC	73558109	IVS-48	Usher syndrome 1d
c.7225-2A>G	73559247	IVS-50	Usher syndrome 1
c.7362+5G>A	73559391	IVS-51	Usher syndrome 1
c.7482+1G>A	73560513	IVS-52	Usher syndrome
c.7660+1G>T	73562833	IVS-53	Usher syndrome 1
c.7660+5G>A	73562837	IVS-53	Hearing loss
c.8064+2T>C	73565756	IVS-55	Usher syndrome 1
c.8722+1delG	73567765	IVS-59	Usher syndrome 1
c.9199-4G>A	73571264	IVS-62	Usher syndrome 1
c.9278+5G>C	73571352	IVS-63	Usher syndrome 1
c.9510+19_9510+25delGGCATCA	73572385	IVS-66	Usher syndrome 1
c.9510+1G>A	73572367	IVS-66	Usher syndrome 1

**Table 2:** In silico analysis using the Alamut program showing the splicing predictor scores and the in vivo and in vitro functional effects of mutations c.6050-9G>A and c.6050-15G>A (our data).

Nucleotide Sequence	Splice Site	Splicing Predictor				Observed In Vivo Splicing Events in Control and Patient (Double Heterozygote)	Observed In Vitro Splicing Events
		SSF (0–100)	Max Ent (0–16)	NNSPLICE (0–1)	Genesplicer (0–15)		
WT	WT acceptor	NR	2.45	NR	NR	WT transcript Skipping of exon 46	WT transcript Skipping of exon 46
c.6050-9 G>A	New acceptor	NR	3.54	NR	1.96	Extension of exon 46 due to the upstream addition of 7nt Skipping of exon 46. WT transcript	New acceptor site: + 7nt intronic included in extended exon 46 Skipping of exon 46
c.6050-15G>A	New acceptor	75.34	6.23	NR	7.84	Extension of exon 46 due to the upstream addition of 13 nt Skipping of exon 46.WT transcript	New acceptor site: + 13nt intronic included in extended exon 46 Skipping of exon 46

NR: not recognized, WT: wild-type. In red- altered splicing scores and aberrant splicing events.

## Supplementary Materials

The following are available online at https://www.mdpi.com/2073-4425/10/10/732/s1, Figure S1: Sanger sequencing of the *CDH23* transcripts detected in vivo.
