# Supplementary material for: Aberrant Splicing Events Associated to CDH23 Noncanonical Splice Site Mutations in a Proband with Atypical Usher Syndrome 1
Source: Genes (Basel). 2019 Sep 21;10(10):732. doi: 10.3390/genes10100732 (PMC6826400; doi:10.3390/genes10100732)
Supplement: Supplementary file 1 [file genes-10-00732-s001.pdf]

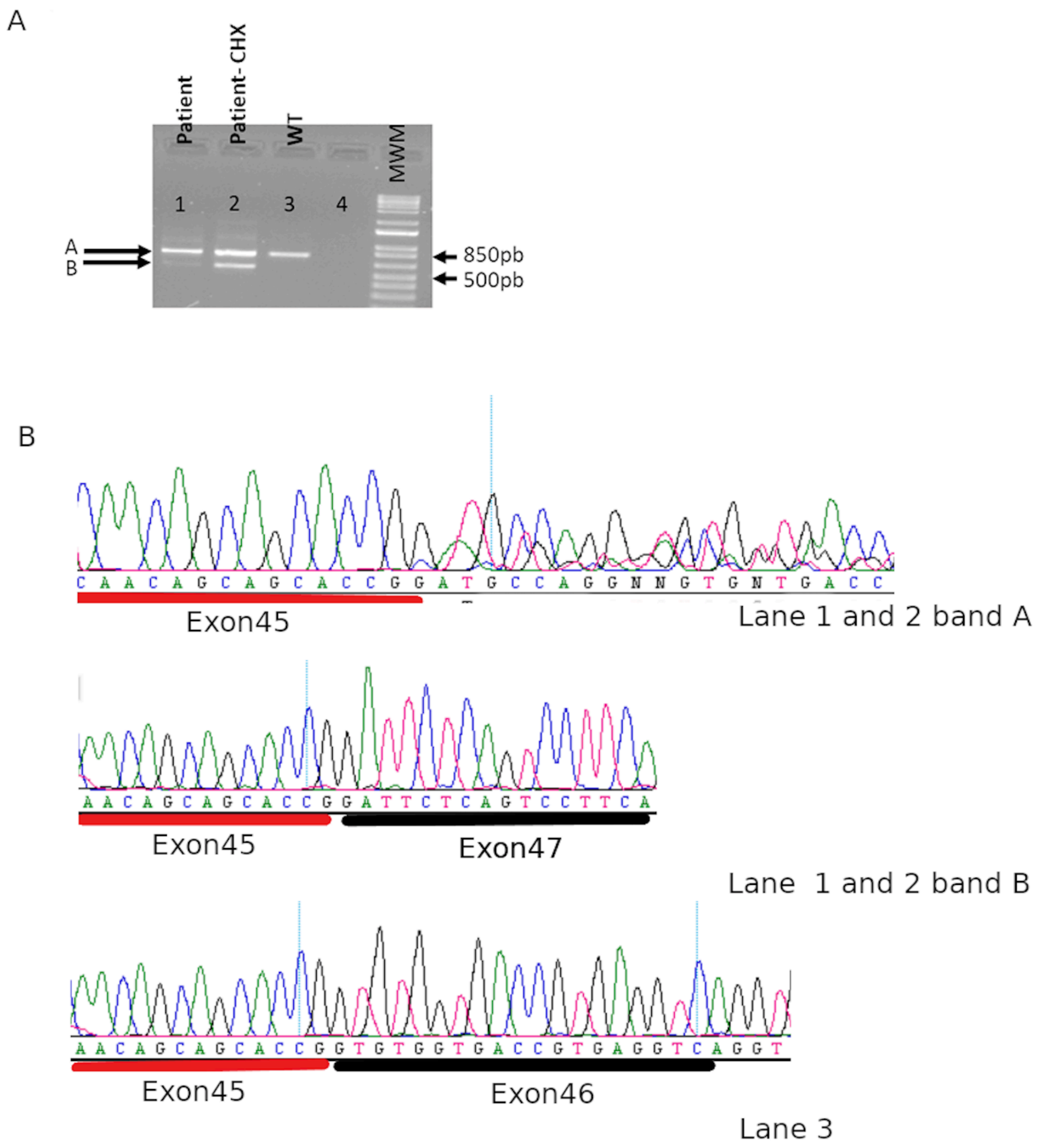

Supplementary Figure 1.- Sanger sequencing of the *CDH23* transcripts detected in vivo. A) Bands amplified by RT-PCR using primers located in exons 45 and 47 of *CDH23* from peripheral blood samples of patient and control (as reported in Figure 3A). Lanes 1 and 2- Untreated and cycloheximide-treated patient sample, respectively); Lane 3- Untreated control sample. **B)** Electropherograms of purified gel bands as indicated. Note that Band A of Lanes 1 and 2 shows triple sequence downstream exon 45 junction, being the result of an admixture of the spliced products WT, +13 and +7 additional nt transcript bands in the patient's sample. Confirmation of these bands was obtained by cloning and Sanger sequencing of single colonies. Among them, approximately 25% of the clones contained the wild-type spliced product, and the rest were aberrant spliced products (around 50% each). Band B of Lanes 1 and 2 is shorter in size and correspond to exon 46 skipping transcript. Lane 3 electropherogram corresponds to the WT spliced product.
